# Supplementary material for: Behavioral and neuro-cognitive bases for emergence of norms and socially shared realities via dynamic interaction
Source: Commun Biol. 2022 Dec 15;5:1379. doi: 10.1038/s42003-022-04329-1 (PMC9754314; doi:10.1038/s42003-022-04329-1)
Supplement: Supplementary file 2 — Supplementary Information [file 42003_2022_4329_MOESM2_ESM.pdf]

Supplementary information for "Behavioral and neuro-cognitive bases for emergence of norms and socially shared realities via dynamic interaction"

Kiri Kuroda†, Yukiko Ogura†, Akitoshi Ogawa, Tomoya Tamei, Kazushi Ikeda, Tatsuya Kameda*

*Corresponding author: tkameda@l.u-tokyo.ac.jp
†These authors contributed equally.

This file includes:

Supplementary Notes 1–3

Supplementary Figs. 1–12

Supplementary Tables 1 and 2

Supplementary Note 1: Exploratory whole-brain analysis

We had an a priori hypothesis that cognitive perspective taking would be associated with the covert-level convergence and stabilization of individual psychophysical functions. Hence, we used the functional localizer with the ToM task to identify the ROIs in the mentalizing network. To assess validity of the results of ROI analyses, we also conducted the following exploratory whole-brain analysis.

First, we searched for the brain region that positively tracked the similarity parameter during the interaction. When interacting with the Sherif-type partner, the bilateral TPJ positively tracked similarity (Supplementary Fig. 8a), but only white matter regions were detected for the Asch-type partner (Supplementary Fig. 8b). Next, we searched for the brain regions in which the neural effect of similarity during interaction covaried with the stabilization of estimation weights after interaction (i.e., the brain regions in which the neural effect of similarity negatively correlated with σ after interaction). The weights in the robust correlation analysis in Fig. 3d were included in the second-level analysis as a nuisance covariate. For the Sherif-type partner, the larger the neural effect of similarity in the RTPJ and the right dorsolateral prefrontal cortex, the greater the stability after interaction (Supplementary Fig. 8c), but for the Asch-type partner, no suprathreshold cluster was detected. Last, we searched for brain regions in which functional connectivity with the RTPJ as a seed region during the interaction covaried with the stabilization of estimation weights (σ) after the interaction. The weights in the robust correlation analysis in Fig. 3e were also included in the second-level analysis as a nuisance covariate. In the brain regions, including the DMPFC and the left TPJ (LTPJ), the larger the functional connectivity with the RTPJ, the greater the stability after the interaction with the Sherif-type partner (Supplementary Fig. 8d), but no suprathreshold cluster was detected for the Asch-type partner. A cluster-forming threshold of $P < .001$ and a cluster-size threshold of $k > 100$ were used for the exploratory whole-brain analyses. The results of

this whole-brain analysis were in line with our a priori hypothesis, confirming that we did not ignore other major activations or overestimate the minor activations in our ROI analyses.

Supplementary Note 2: Attention to partners' estimates

To check whether participants paid attention to the computer partner's estimate after each trial, we recorded participants' eye movements while the estimates were presented. Five participants were excluded from the analysis because the pupils were often hidden by the coil when these participants looked at the right side of the monitor. The threshold of fixation duration was set to 50 ms. Each fixation was classified as "self" or "partner" if participants looked more than 5 visual degrees left or right from the center of the monitor, respectively. The analysis showed that participants first looked at the partners' estimates more frequently than their own estimates [Supplementary Fig. 9; Sherif-type partner: paired *t* test: $t_{(22)} = 4.31$, $P < .001$; Asch-type partner: paired *t* test: $t_{(22)} = 2.78$, $P = .011$].

Supplementary Note 3: Post-session questionnaire

After scanning, participants answered five items about the experiment on 7-point Likert scales (we asked the same set of five questions in the online behavioral experiment as well):

Q1. When you performed the task with Person A/B, were you confident about your estimates? (1 = *Not confident at all*, 7 = *Very confident*)

Q2. Who do you think gave more accurate estimates, you or Person A/B? (1 = *Certainly me*, 7 = *Certainly Person A/B*)

Q3. Do you feel that your way of estimating the number of dots changed while you performed the task with Person A/B? (1 = *Not at all*, 7 = *Very much*)

Q4. Do you feel that Person A/B's way of estimating the number of dots changed while you performed the task with Person A/B? (1 = *Not at all*, 7 = *Very much*)

Q5. Do you feel that you and Person A/B came to give more similar estimates as you performed the task with Person A/B? (1 = *Not at all*, 7 = *Very much*).

As seen in Supplementary Fig. 10, the Wilcoxon signed-rank test yielded significant differences between the Sherif-type and Asch-type partners for Q2 (Z = 3.08, P = .001. r = 0.58), Q3 (Z = 2.08, P = .038, r = 0.39), and Q5 (Z = 2.34, P = .018, r = 0.44). To summarize these patterns, we further conducted a factor analysis on all five items. The Kaiser–Meyer–Olkin measure of sampling adequacy was 0.67 for the fMRI experiment and 0.65 for the online experiment; both values are above the recommended value of 0.6. For both the fMRI and online experiments, Bartlett's test of sphericity was also significant [fMRI: $\chi^2_{(10)}$ = 79.05, P < .001; online: $\chi^2_{(10)}$ = 210.33, P < .001). The parallel analyses further suggested that a two-factor solution was most appropriate for both experiments. We thus performed the maximum-likelihood factor analysis with two factors using the promax rotation for the factor-loading matrix. The factor analysis identified one factor with a high loading on Q3 (fMRI: 0.35; online: 0.56), Q4 (fMRI: 0.60; online: 0.63), and Q5 (fMRI: 1.00; online: 0.95), which we interpreted to reflect participants' subjective feeling about convergence of their inner (generative) model with that of a partner. As seen in Supplementary Fig. 11, the factor score was significantly different between the two computer partners [fMRI: $t_{(27)}$ = –2.23, P = .034; online: $t_{(212.58)}$ = –3.38, P = .001], indicating that participants felt greater convergence with the Sherif-type than with the Asch-type partner subjectively (although they were actually influenced to the same extent by both types: Fig. 2b).

Supplementary Fig. 1 Linear relationships between the number of dots and participants' dot estimation in Phase 1 of the laboratory behavioral experiment.

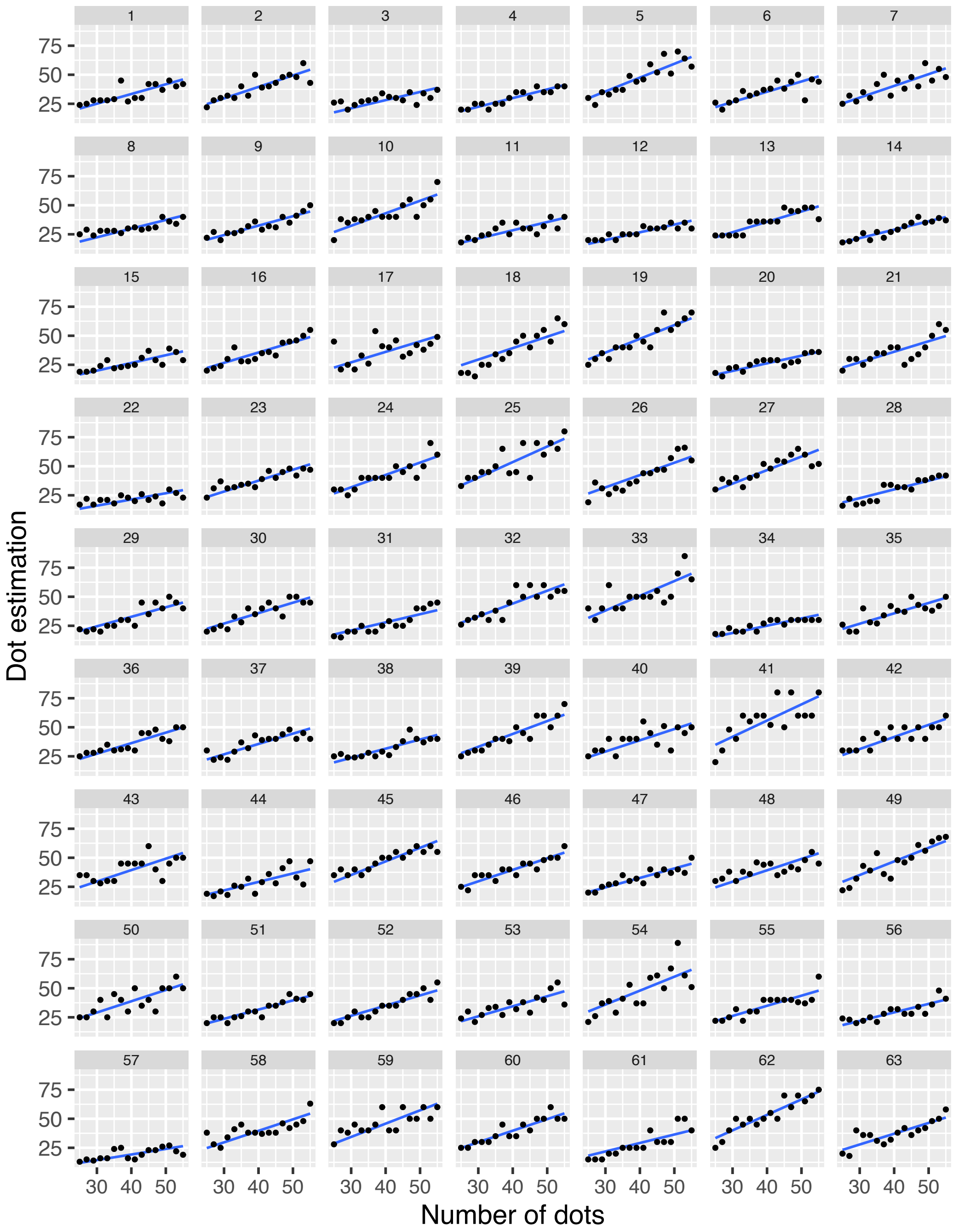

Each panel indicates one participant's data. The linear model (blue regression line) captures participants' estimation well. See also Supplementary Table 1 for the results of model comparison against the log-linear model.

Supplementary Fig. 2 Absolute differences in estimation weights between participants in real and nominal pairs in Phases 1 and 3.

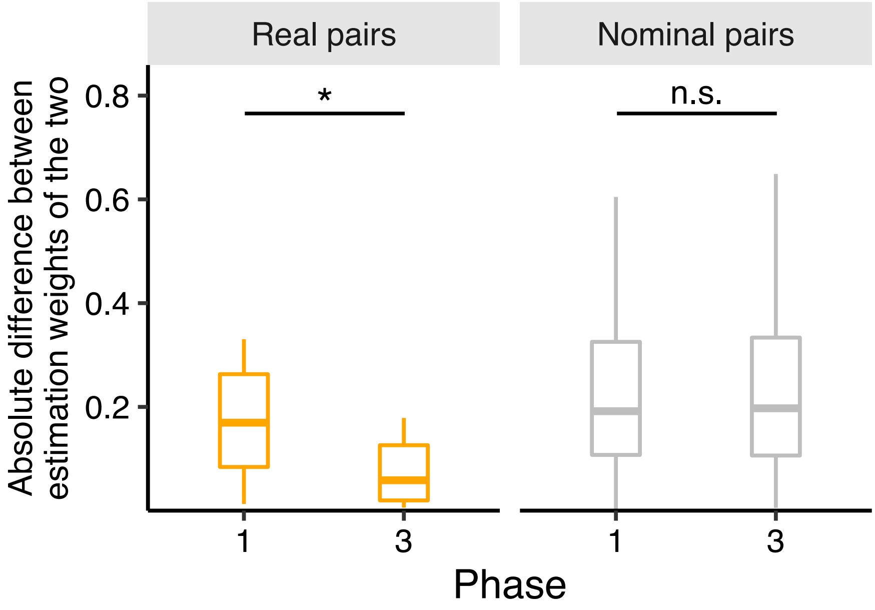

The 210 nominal pairs were generated by combining the 21 participants in the individual condition in a brute-force fashion. No significant change was found between the phases in the nominal pairs [paired *t* test: $t_{(209)} = 1.10$, $P = .275$], and the decrease was larger in the real pairs [Welch's *t* test: $t_{(24.1)} = 3.01$, $P = .006$]. The box plots indicate the medians, the first and third quartiles, and the values no further than 1.5 inter-quartile range from the quartiles. *$P < .05$.

Supplementary Fig. 3 Negative correlations between the difference in σ and the difference in Kendall's τ from the pre- to the post-interaction phase.

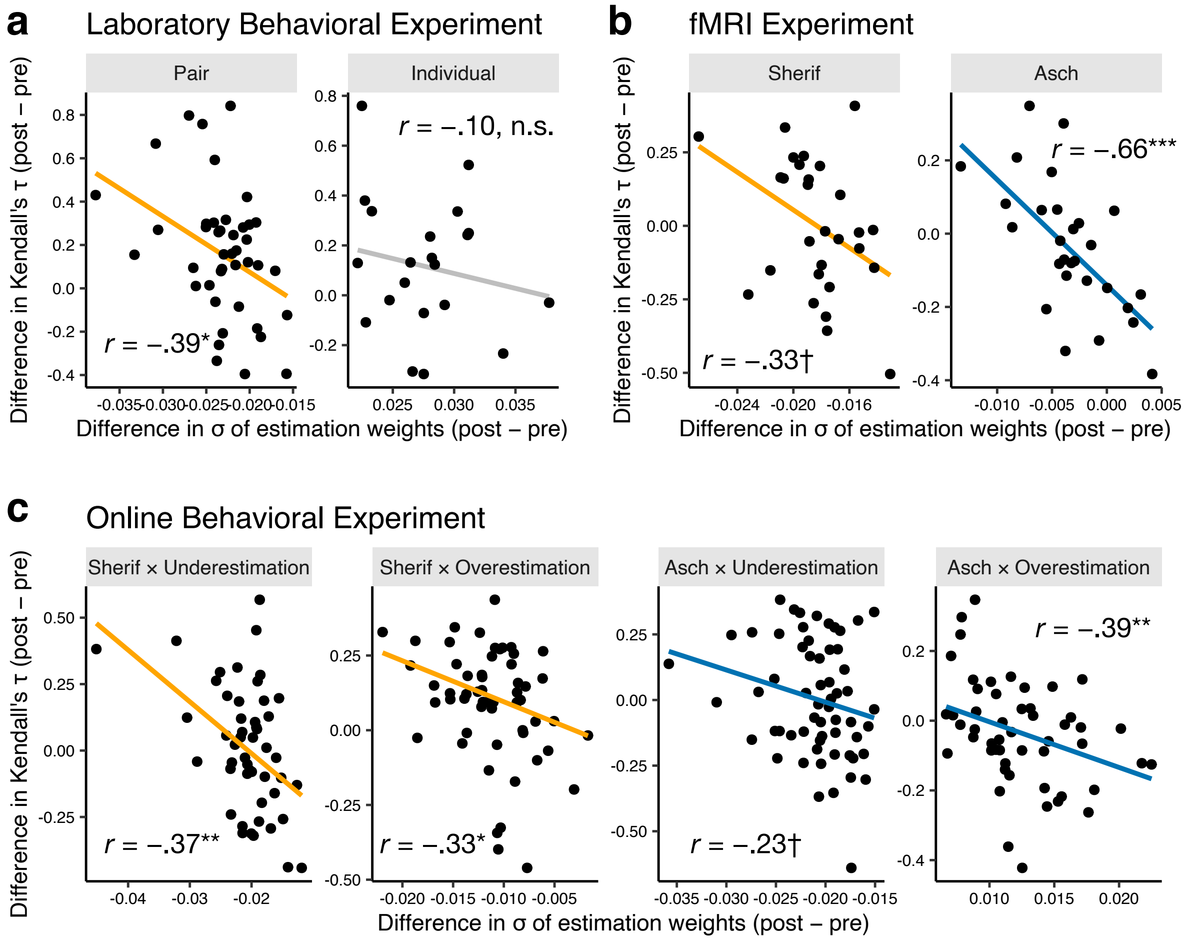

a, **b**, and **c** show the data from the laboratory, fMRI, and online experiments, respectively. Smaller σ indicates that the participant's estimation weights were more stable. Kendall's τ (Fisher z transformed) indicates the rank correlation between the true and estimated number of dots. Thus, negative correlations between these two indices mean that participants whose estimations were stabilized more after interaction (greater decrease in σ) also showed greater improvement in accuracy in relative ordering of the stimuli. Each dot corresponds to one participant's data, and each panel displays one condition in each experiment. The bold line in each panel is the linear regression line. Robust correlation coefficient r is displayed in each panel. †$P < .10$, *$P < .05$, **$P < .01$, ***$P < .001$.

Supplementary Fig 4. Timelines of the dot-estimation task in the fMRI experiment.

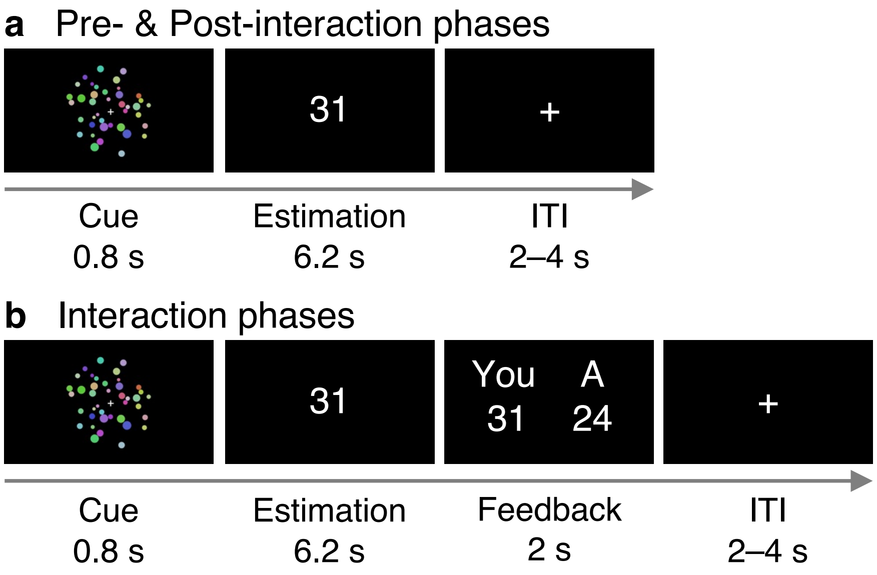

a In the pre- and post-interaction phases, participants performed the dot-estimation task individually. **b** In the interaction phases, participants were each paired with a computer partner ("A"); both observed the same dots and estimated the number of dots independently, and then their estimates were presented to each other every trial. ITI = Intertrial interval.

Supplementary Fig 5. Widely applicable information criteria (WAIC) of the full model and seven reduced models.

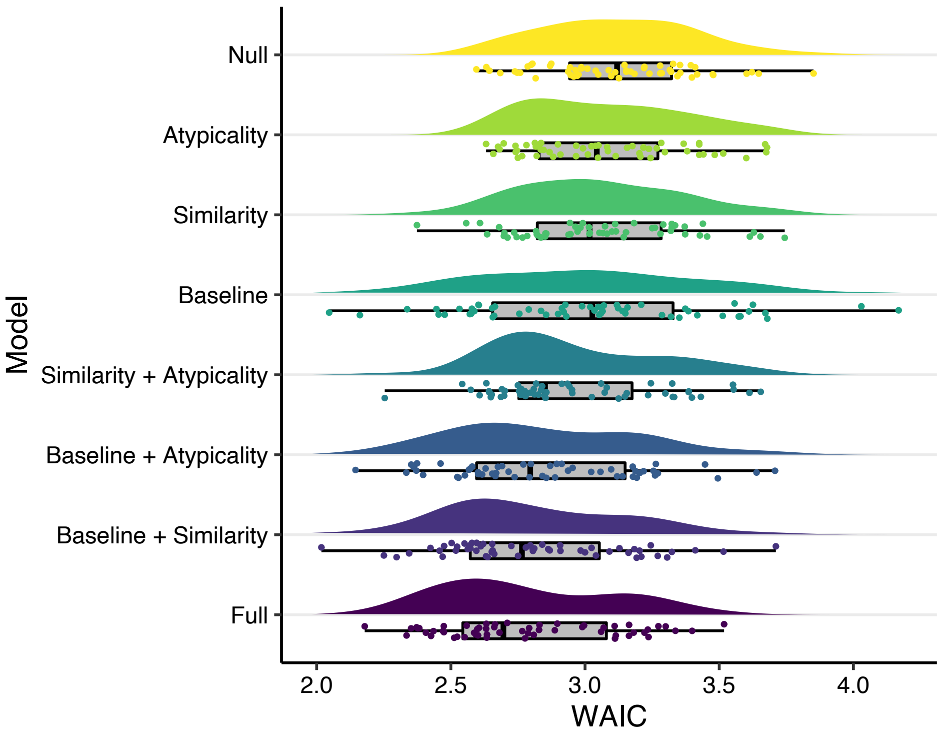

The full model outperformed the others in terms of prediction. Each dot indicates one participant's data. The box plots indicate the medians, the first and third quartiles, and the values no further than 1.5 inter-quartile range from the quartiles.

Supplementary Fig 6. Posterior predictive check of our model (Eqs. 11–13).

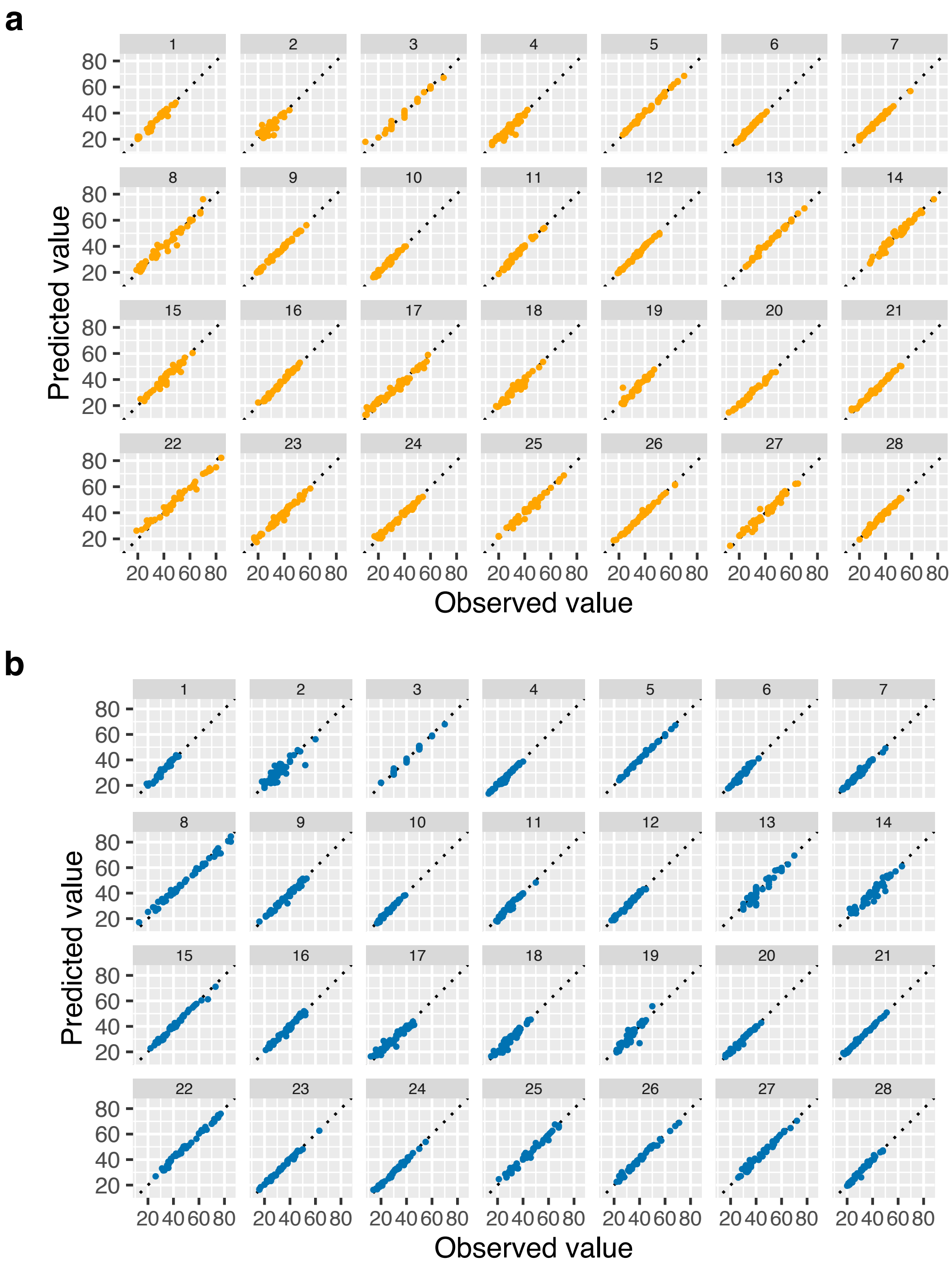

There were strong positive correlations between the observed and predicted estimates of the number of dots in each trial in the interaction phases with the Sherif-type partner (**a**: *r*s > .84) and the Asch-type partner (**b**: *r*s > .89). Each panel displays one participant's data.

Supplementary Fig 7. Group results from the functional localizer task for cognitive perspective taking.

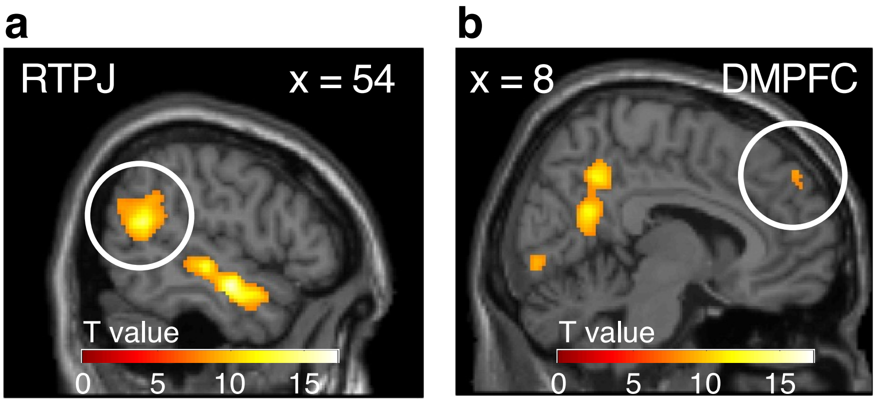

The peaks of the right temporoparietal junction (**a**: RTPJ) and dorsomedial prefrontal cortex (**b**: DMPFC) are highlighted.

Supplementary Fig 8. Results of the exploratory whole-brain analysis.

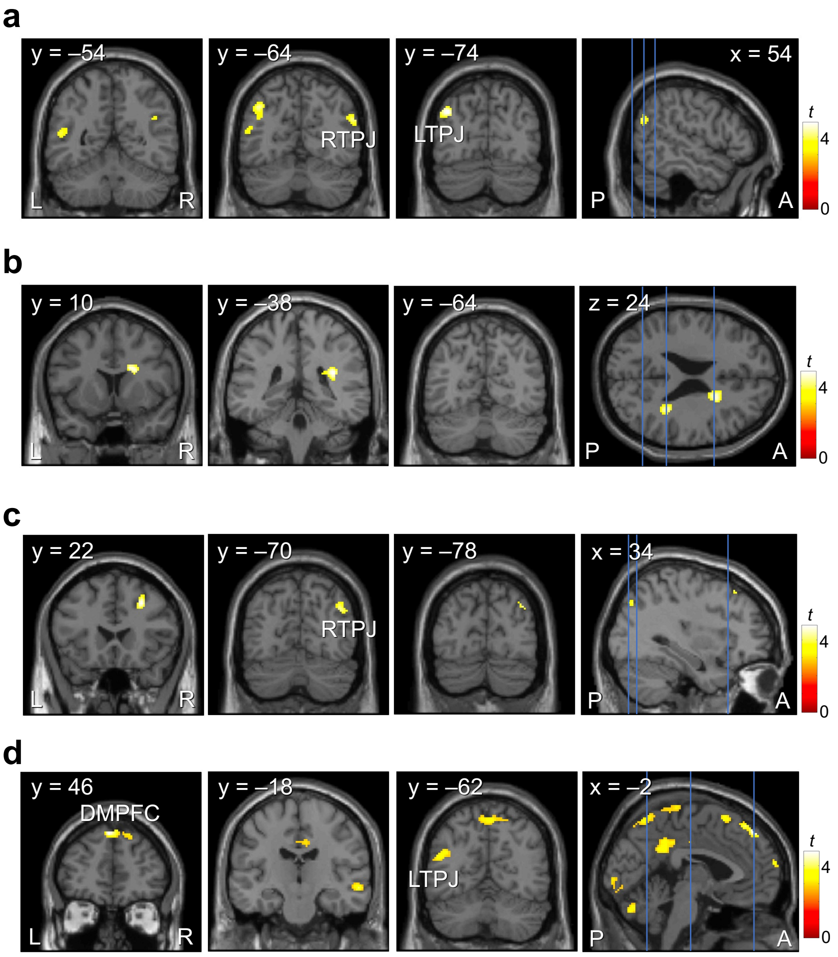

Brain regions that positively tracked the *Similarity* parameter during interaction with (**a**) the Sherif-type and (**b**) the Asch-type partners. **c** Brain regions in which the neural effect of similarity during the interaction covaried with the stabilization of estimation weights after interaction with the Sherif-type partner. For the Asch-type partner, no suprathreshold cluster was detected. **d** Brain regions in which functional connectivity with the right temporoparietal junction (RTPJ) as a seed region during the interaction covaried with the stabilization of estimation weights after the interaction with the Sherif-type partner. For the Asch-type partner, no suprathreshold cluster was detected. DMPFC = Dorsomedial prefrontal cortex; LTPJ = left temporoparietal junction.

Supplementary Fig 9. The eye-tracking data from the fMRI experiment.

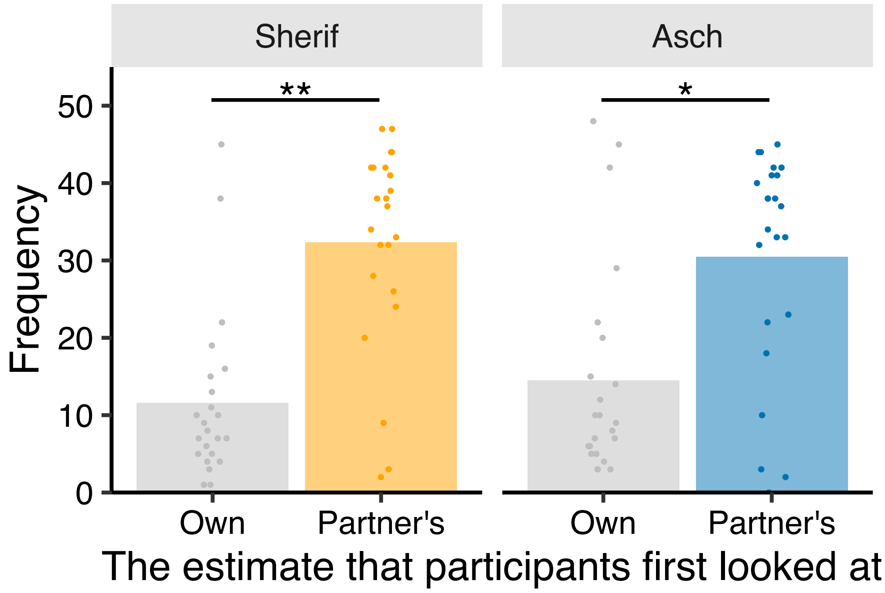

Participants first looked at the partner's estimate more frequently than their own estimate. The bar plots indicate the means across participants. *$P < .05$, **$P < .01$.

Supplementary Fig 10. Results from the post-session questionnaire.

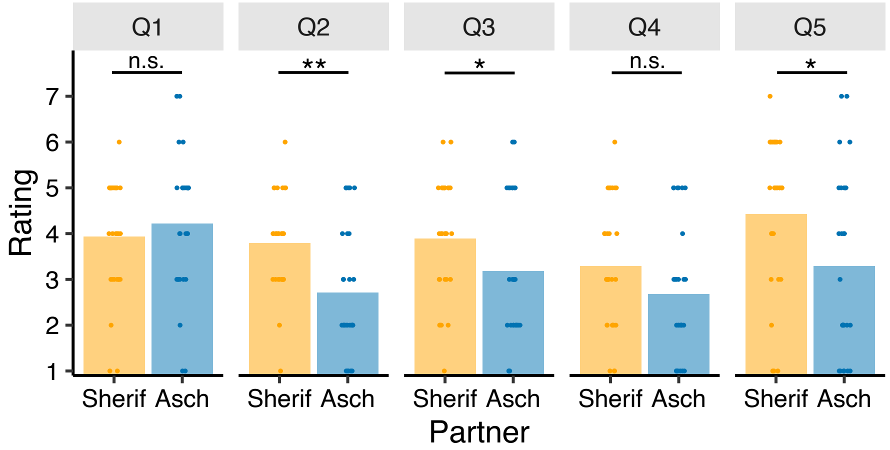

See Supplementary Note 3 for details. The bar plots indicate the means across participants. *$P < .05$, **$P < .01$.

Supplementary Fig 11. Results from the factor analysis of the post-session questionnaire.

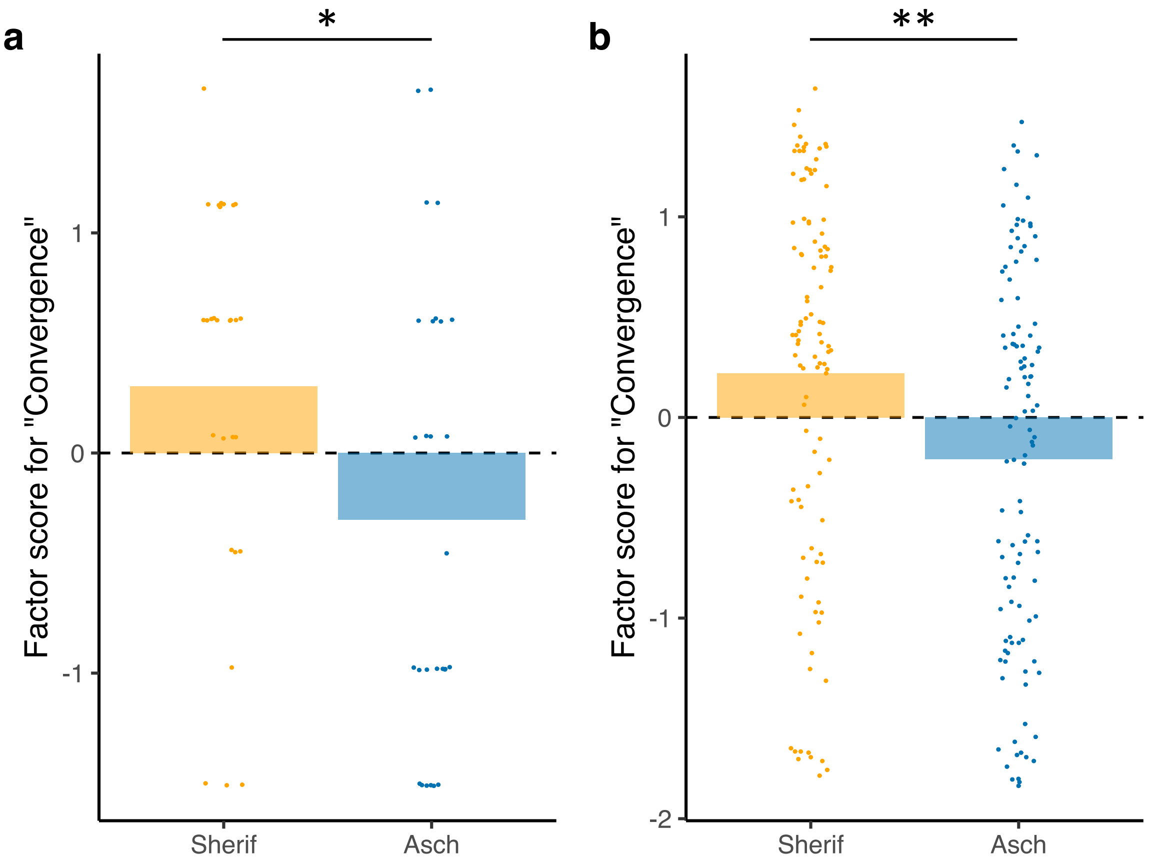

a: the fMRI experiment; **b**: the online behavioral experiment. The factor scores of "subjective convergence of the inner model" with a partner, when participants interacted with the Sherif-type and the Asch-type agent, are shown respectively. See Supplementary Note 3 for details about the factor analysis. The bar plots indicate the means across participants. *$P < .05$, **$P < .01$.

Supplementary Fig 12. Participants' estimation weights (w_i) in the four conditions in the online behavioral experiment.

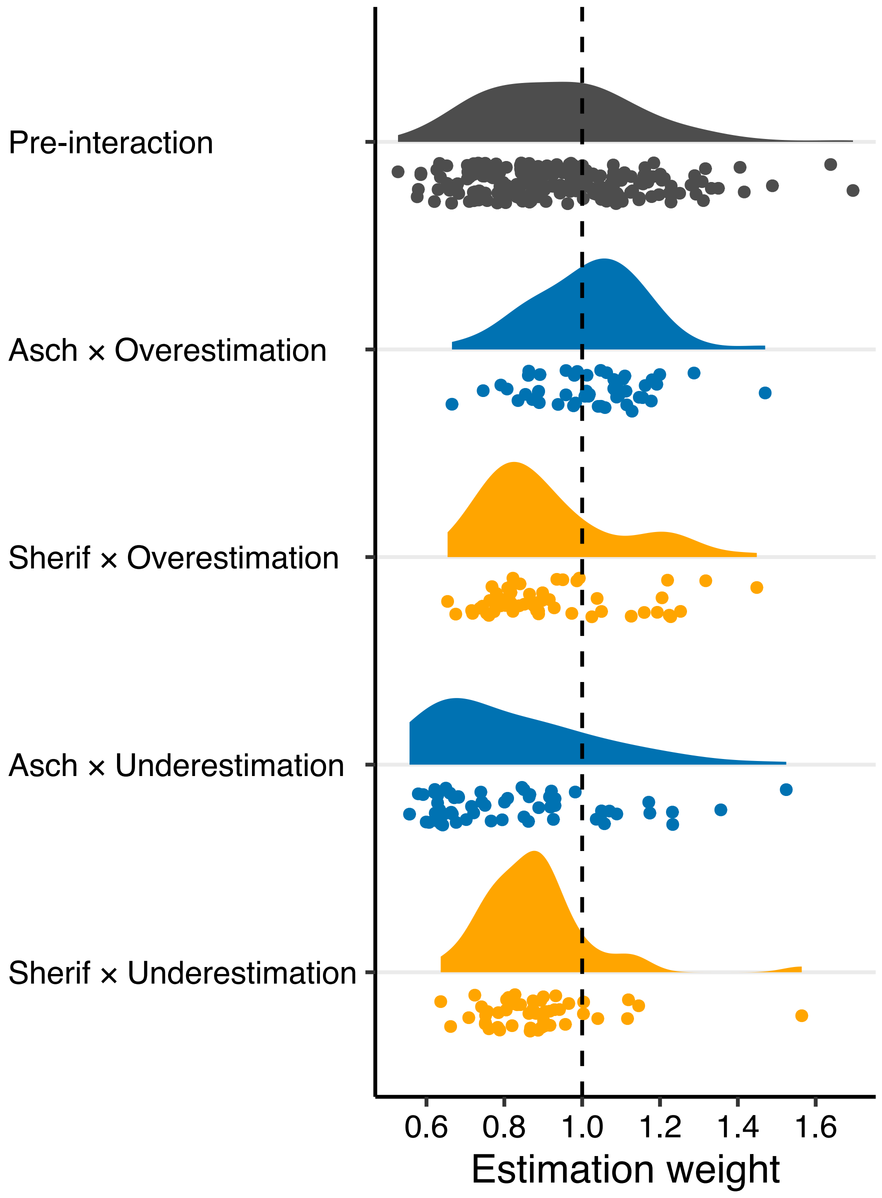

The experiment used a 2 × 2 between-participants design, with factors partner type (Sherif vs. Asch) and the built-in estimation bias of the partner (underestimation vs. overestimation). Each point indicates one participant's estimation weight.

Supplementary Table 1. Results from the linear and log-linear psychophysical models for the dot-estimation task (mean and SEM across participants).

Experiment	Condition/Phase	Linear model		Log-linear model	
		w	AIC	*w*	AIC
Laboratory behavioral	Pair	0.91 (0.03)	97 (1.7)	10.11 (0.33)	109 (2.1)
	Individual	0.96 (0.04)	102 (2.5)	10.66 (0.48)	113 (2.4)
fMRI	Pre-interaction	0.96 (0.04)	159 (3.3)	10.81 (0.42)	181 (2.9)
	Sherif	0.87 (0.03)	149 (2.8)	9.89 (0.30)	166 (2.8)
	Asch	0.84 (0.03)	145 (2.9)	9.52 (0.38)	162 (2.9)
	Post-Sherif	0.89 (0.03)	150 (2.9)	10.09 (0.35)	169 (2.3)
	Post-Asch	0.86 (0.04)	149 (2.6)	9.78 (0.42)	163 (3.2)
Online behavioral	Sherif/Under	0.90 (0.03)	152 (2.0)	10.29 (0.28)	170 (2.3)
	Sherif/Over	0.92 (0.02)	156 (2.1)	10.49 (0.27)	174 (2.1)
	Asch/Under	0.87 (0.03)	152 (2.3)	9.87 (0.29)	169 (2.3)
	Asch/Over	0.98 (0.02)	157 (1.9)	11.22 (0.23)	177 (1.9)

Note. AIC = Akaike information criterion; fMRI = functional magnetic resonance imaging; Over = overestimation; Under = underestimation.

Supplementary Table 2. Group-level results from the localizer task for cognitive perspective taking.

Contrast / Regions	MNI coordinates of the peak (mm)			T value (peak)	p-FWE (cluster)	Number of voxels
	x	*y*	*z*			
ToM vs. non-ToM						
RTPJ	54	−54	18	13.9	< .001	1039
LTPJ	−54	−60	16	11.1	< .001	466
DMPFC	8	52	44	8.52	< .001	17
Precuneus	6	−58	24	13.0	< .001	1072

Note. ToM = Theory of mind; RTPJ = right temporoparietal junction; LTPJ = left temporoparietal junction; DMPFC = dorsomedial prefrontal cortex.
