# Supplementary material for: Behavioral and neuro-cognitive bases for emergence of norms and socially shared realities via dynamic interaction
Source: Commun Biol. 2022 Dec 15;5:1379. doi: 10.1038/s42003-022-04329-1 (PMC9754314; doi:10.1038/s42003-022-04329-1)
Supplement: Supplementary file 3 — Reporting Summary [file 42003_2022_4329_MOESM3_ESM.pdf]

Corresponding author(s): Tatsuya Kameda

Last updated by author(s): Nov 4, 2022

Reporting Summary

Nature Portfolio wishes to improve the reproducibility of the work that we publish. This form provides structure for consistency and transparency in reporting. For further information on Nature Portfolio policies, see our Editorial Policies and the Editorial Policy Checklist.

Statistics

For all statistical analyses, confirm that the following items are present in the figure legend, table legend, main text, or Methods section.

n/a	Confirmed	
☐	☒	The exact sample size (*n*) for each experimental group/condition, given as a discrete number and unit of measurement
☐	☒	A statement on whether measurements were taken from distinct samples or whether the same sample was measured repeatedly
☐	☒	The statistical test(s) used AND whether they are one- or two-sided<br>*Only common tests should be described solely by name; describe more complex techniques in the Methods section.*
☒	☐	A description of all covariates tested
☐	☒	A description of any assumptions or corrections, such as tests of normality and adjustment for multiple comparisons
☐	☒	A full description of the statistical parameters including central tendency (e.g. means) or other basic estimates (e.g. regression coefficient) AND variation (e.g. standard deviation) or associated estimates of uncertainty (e.g. confidence intervals)
☐	☒	For null hypothesis testing, the test statistic (e.g. *F*, *t*, *r*) with confidence intervals, effect sizes, degrees of freedom and *P* value noted<br>*Give P values as exact values whenever suitable.*
☐	☒	For Bayesian analysis, information on the choice of priors and Markov chain Monte Carlo settings
☐	☒	For hierarchical and complex designs, identification of the appropriate level for tests and full reporting of outcomes
☐	☒	Estimates of effect sizes (e.g. Cohen's *d*, Pearson's *r*), indicating how they were calculated

Our web collection on statistics for biologists contains articles on many of the points above.

Software and code

Policy information about availability of computer code

Data collection	Matlab, Psychtoolbox 3, Psychopy 2 on Windows 10, 3T Siemens Prisma
Data analysis	Matlab R2017a, SPM12(v7219), MarsBaR Toolbox (v0.44), R (v3.6.1), Stan (v2.21.2)

For manuscripts utilizing custom algorithms or software that are central to the research but not yet described in published literature, software must be made available to editors and reviewers. We strongly encourage code deposition in a community repository (e.g. GitHub). See the Nature Portfolio guidelines for submitting code & software for further information.

Data

Policy information about availability of data

All manuscripts must include a data availability statement. This statement should provide the following information, where applicable:

- Accession codes, unique identifiers, or web links for publicly available datasets
- A description of any restrictions on data availability
- For clinical datasets or third party data, please ensure that the statement adheres to our policy

The data analyzed in this study are available for download: https://osf.io/tfy9s/

Human research participants

Policy information about studies involving human research participants and Sex and Gender in Research.

Reporting on sex and gender	See "Research sample" below for the number of male/female participants. We did not perform any sex-based analysis.
Population characteristics	See "Research sample" below.
Recruitment	Participants were recruited from the University of Tokyo student population. The participant pool is maintained by the Social Psychology Laboratory. There were no apparent biases in recruitment that would impact the current findings. All participants were incentivized to perform best for their own rewards in the experiment.
Ethics oversight	The Ethics Committee of the Department of Social Psychology at the University of Tokyo

Note that full information on the approval of the study protocol must also be provided in the manuscript.

Field-specific reporting

Please select the one below that is the best fit for your research. If you are not sure, read the appropriate sections before making your selection.

☐ Life sciences ☒ Behavioural & social sciences ☐ Ecological, evolutionary & environmental sciences

For a reference copy of the document with all sections, see nature.com/documents/nr-reporting-summary-flat.pdf

Behavioural & social sciences study design

All studies must disclose on these points even when the disclosure is negative.

Study description	Quantitative experimental study.
Research sample	Laboratory behavioral experiment: 63 students at the University of Tokyo (pair condition: 27 men and 15 women; 21.2 ± 1.0 years; individual condition: 14 men and 7 women; 22.0 ± 1.7 years). fMRI Experiment: 28 right-handed students at the University of Tokyo (13 men and 15 women; 22.2 ± 2.3 years) with no history of neurological or psychiatric illness and no overlap with participants in the behavioral experiment. Online behavioral experiment: 216 students at the University of Tokyo with no overlap with participants in the other experiments (Sherif × Underestimation: n = 49; Sherif × Overestimation: n = 56; Asch × Underestimation: n = 60; Asch × Overestimation: n = 51)
Sampling strategy	In the laboratory behavioral experiment, sample size was determined by a power analysis on findings from a previous study employing a similar experimental paradigm (Mutata, Nishida, Watanabe & Kameda, 2020, "Convergence of physiological responses to pain during face-to-face interaction", Scientific Reports, 10:450). In the fMRI experiment, we conducted a power analysis assuming a two-tailed paired t-test with $\alpha = 0.05$, $\beta = 0.8$, and Cohen's d = 0.6, using G*power (http://www.psychologie.hhu.de/arbeitsgruppen/allgemeine-psychologie-und-arbeitspsychologie/gpower.html). From this analysis, we found that 24 or more participants were needed in the fMRI experiment. For the online behavioral experiment, we pre-registered the sample size before conducting the experiment.
Data collection	Data was collected at the Center for Evolutionary Cognitive Sciences at the University of Tokyo using a Siemens Prisma 3T MRI scanner. Stimulus timing and behavioral data collection was computer based.
Timing	Laboratory behavioral experiment: April 1–8, 2016<br>fMRI experiment: December 19, 2017–February 2, 2018<br>Online behavioral experiment: July 2–4, 2021
Data exclusions	In the fMRI experiment, we excluded 11 trials from analysis because the participant's estimate was incomplete.
Non-participation	In the fMRI experiment, we excluded 2 participants who did not follow the experimental instruction, before analyzing the data.
Randomization	All participants were randomly assigned to the experimental conditions.

Reporting for specific materials, systems and methods

We require information from authors about some types of materials, experimental systems and methods used in many studies. Here, indicate whether each material, system or method listed is relevant to your study. If you are not sure if a list item applies to your research, read the appropriate section before selecting a response.

Materials & experimental systems

n/a	Involved in the study
☒	☐ Antibodies
☒	☐ Eukaryotic cell lines
☒	☐ Palaeontology and archaeology
☒	☐ Animals and other organisms
☒	☐ Clinical data
☒	☐ Dual use research of concern

Methods

n/a	Involved in the study
☒	☐ ChIP-seq
☒	☐ Flow cytometry
☐	☒ MRI-based neuroimaging

Magnetic resonance imaging

Experimental design

Design type

Event-related design

Design specifications

The experiment consisted of one pre-interaction phase (block), two interaction phases, and two post-interaction phases (24, 48, and 24 trials, respectively). First, all participants performed the estimation task individually in the pre-interaction phase. Participants were then paired with either the "Sherif-type" or the "Asch-type" partner for the interaction phase. Completing the interaction phase, participants proceeded to the post-interaction phase and worked on the estimation task again individually. Before proceeding to the second interaction phase, participants worked on the Theory-of-Mind localizer task to be used as a functional localizer. After that, for the second interaction phase, participants were paired with the other computer partner (the order of two types of partners was counterbalanced across participants). Finally, participants worked on the estimation task individually again in the second post-interaction phase.

Behavioral performance measures

Participants estimated the number of dots, which were shown on a computer screen briefly, by button pressing.

Acquisition

Imaging type(s)

Functional and structural

Field strength

3T

Sequence & imaging parameters

Functional images were obtained using a multiband EPI sequence provided by the Center for Magnetic Resonance Research, the University of Minnesota (Release R016). The scanning parameters for the EPI were as follows: TR: 1000 ms; TE: 30 ms; FOV: 216 × 216 mm2; matrix: 72 × 72, resolution: 3 × 3 × 3 mm2; 45 slices with no gap; flip angle: 59°; multiband factor: 3.

Area of acquisition

Whole brain

Diffusion MRI ☐ Used ☒ Not used

Preprocessing

Preprocessing software

SPM12(v7219)

Normalization

We performed (i) slice-timing correction using the first slice as a reference, (ii) spatial realignment, (iii) coregistration of structural and functional images, (iv) spatial normalization to the Montreal Neurological Institute space, and (v) spatial smoothing (full-width at half-maximum of Gaussian kernel = 8 mm isotropic). Low frequency noise was removed by a high-pass filter of 128 seconds.

Normalization template

The Montreal Neurological Institute (MNI) template

Noise and artifact removal

To remove the effects of head movement and physiological noise, we included nine nuisance regressors (translations along the x-, y-, and z-axis, rotations of pitch, roll, and yaw, heart rate, respiration, and DVARS) in each GLM. To construct a heart rate regressor, we identified the peaks in the 6-s window in the pulse wave signal and obtained the inverse number of the average peak-to-peak duration. To construct a respiratory regressor, we calculated the standard deviation of respiration signal in the 6-s window for each TR.

Volume censoring

No volume censoring was performed.

Statistical modeling & inference

Model type and settings

Mass univariate; Three types of general linear models (GLMs) were adopted for each participant in the interaction runs. GLM1 examined the brain regions that tracked Similarity in the time-series model (Eq. S9). GLM1 included two condition regressors for Cue and Feedback as events (see Fig. S3 bottom). For the Cue regressor, we included five parametric modulators (Baseline, CoefSim(t), Sim(t − 1), CoefAtyp (t), and Atyp(t − 1)), which were derived from the time-series model for each participant, in the design matrix. For the Feedback regressor, two parametric modulators (Sim(t), Atyp(t)) were

included. Each parametric modulator was scaled by z-score normalization. GLM2 examined the functional connectivity related to Similarity. GLM2 also included two condition regressors for Cue and Feedback as events. For the Cue regressor, Similarity (Sim(t − 1)) regressor was included. GLM3 included two condition regressors for Cue and Feedback as events. For the Cue regressor, the estimated dot number for trial t was included.

Effect(s) tested

The central effect we tested was whether the RTPJ addressed by the Theory-of Mind localizer tracked similarity between participants and the Sherif-type partner.

Specify type of analysis: ☐ Whole brain ☒ ROI-based ☐ Both

Anatomical location(s)

Using the Theory-of-Mind localizer task, ROIs in the RTPJ (group peak = [54 –54 18]) and the DMPFC (group peak = [8 52 44]) were addressed. The local peak nearest to the group peak (Table S2) was identified individually for each participant for the RTPJ ROI and for 26 of 28 participants for the DMPFC ROI (we substituted the group peak of DMPFC for the remaining two participants). Both ROIs for each participant were a 6-mm radius sphere centered on the respective individual peak defined using MarsBaR toolbox (ver. 0.44).

Statistic type for inference
(See Eklund et al. 2016)

Voxel-wise inference

Correction

FWE correction was conducted when necessary.

Models & analysis

n/a	Involved in the study
☐	☒ Functional and/or effective connectivity
☒	☐ Graph analysis
☒	☐ Multivariate modeling or predictive analysis

Functional and/or effective connectivity

We conducted a generalized form of context-dependent psychophysiological interaction (gPPI) analysis.
